# A machine learning approach to classifying New York Heart Association (NYHA) heart failure

**DOI:** 10.1038/s41598-024-62555-5

**Published:** 2024-05-20

**Authors:** Krystian Jandy, Pawel Weichbroth

**Affiliations:** 1 Gdansk University of Technology, Gdańsk, Poland; 2 Department of Software Engineering, Faculty of Electronics, Telecommunications and Informatics, Gdansk University of Technology, Gdańsk, Poland

**Keywords:** NYHA, Classification, Decision-Tree, Interventional cardiology, Risk factors

## Abstract

According to the European Society of Cardiology, globally the number of patients with heart failure nearly doubled from 33.5 million in 1990 to 64.3 million in 2017, and is further projected to increase dramatically in this decade, still remaining a leading cause of morbidity and mortality. One of the most frequently applied heart failure classification systems that physicians use is the New York Heart Association (NYHA) Functional Classification. Each NYHA class describes a patient’s symptoms while performing physical activities, delivering a strong indicator of the heart performance. In each case, a NYHA class is individually determined routinely based on the subjective assessment of the treating physician. However, such diagnosis can suffer from bias, eventually affecting a valid assessment. To tackle this issue, we take advantage of the machine learning approach to develop a decision-tree, along with a set of decision rules, which can serve as additional blinded investigator tool to make unbiased assessment. On a dataset containing 434 observations, the supervised learning approach was initially employed to train a Decision Tree model. In the subsequent phase, ensemble learning techniques were utilized to develop both the Voting Classifier and the Random Forest model. The performance of all models was assessed using 10-fold cross-validation with stratification.The Decision Tree, Random Forest, and Voting Classifier models reported accuracies of 76.28%, 96.77%, and 99.54% respectively. The Voting Classifier led in classifying NYHA I and III with 98.7% and 100% accuracy. Both Random Forest and Voting Classifier flawlessly classified NYHA II at 100%. However, for NYHA IV, Random Forest achieved a perfect score, while the Voting Classifier reported 90%. The Decision Tree showed the least effectiveness among all the models tested. In our opinion, the results seem satisfactory in terms of their supporting role in clinical practice. In particular, the use of a machine learning tool could reduce or even eliminate the bias in the physician’s assessment. In addition, future research should consider testing other variables in different datasets to gain a better understanding of the significant factors affecting heart failure.

## Introduction

American Heart Association, a nonprofit organization founded in 1905 and with headquarters in Dallas (Texas), introduced in 1918 a system of classifying the extent of heart failure, based on patient limitations during physical activity, now known as the New York Heart Association (NYHA) Functional Classification. Currently, NYHA is claimed to be the most commonly applied descriptor of heart failure, also used clinically to determine trial eligibility^2^.

In a typical scenario, NYHA class can take one of four categories, based on the patient’s medical history, physical examination, and assessment of symptoms and functional capacity. A physician considers factors such as the patient’s ability to perform daily activities, exercise tolerance, and the presence of symptoms like shortness of breath, fatigue, and edema (swelling). Based on this assessment, a physician assigns the appropriate NYHA class to the patient, later used to foretell and monitor the effectiveness of treatment interventions^10^.

Nevertheless, as one can already notice the NYHA class is a subjective assessment and can vary depending on the physician’s judgment. Moreover, like any other method is still vulnerable to human error, even though, by assumption, a class is determined through a comprehensive evaluation. In order to address these issues, we employ a machine learning approach to develop a NYHA class predictive model in the form of a decision-tree. Since our model is trained and tested on factual data, it is not influenced by subjective factors, biases, or emotions that can impact human judgment^37^.

The rest of the paper is structured as follows. In section 2, we briefly elaborate on the research topic, as well as on the related work. In section 3 we outline the research methodology, in particular the data sample used, the target attribute, and the preprocessing and classification methods applied. In section 4, we review the results obtained, followed by a discussion in section 5, devoted to the contributions and limitations of the study. In section 6, we conclude the paper.

## Background and related work

According to American Heart Association, heart failure (HF) is “a lifelong condition in which the heart muscle can’t pump enough blood to meet the body’s needs for blood and oxygen”^3^. This prevalent medical condition has been affecting a significant number of individuals worldwide, globally estimated on over 64.3 million people (8.52 per 1,000 inhabitants)^9^. The prevalence of HF appears significantly higher for women, whereas the years lost due to disability is significantly higher for men^24^. In a global perspective, HF still remains a serious public health problem, with elevated morbidity and mortality^14^.

The NYHA classification system is based on the patient’s self-report of symptoms and signs. Patients can move between classes, either down (improvement) or up (deterioration), depending on the severity of their disease at the time. The NYHA system was designed for clinical assessment of patients by physicians, and termed by the four NYHA classes marked by Roman numerals, which correspond to the patient’s limitations in physical activities caused by cardiac symptoms. Table 1 shows the current version of the NYHA classification system.

**Table 1:** The 1994 New York Heart Association (NYHA) Classification. Source:^29^.

Class	Symptoms
I	No limitation of physical activity. Ordinary physical activity does not cause undue fatigue, palpitation or shortness of breath
II	Slight limitation of physical activity. Comfortable at rest. Ordinary physical activity results in fatigue, palpitation, shortness of breath or chest pain
III	Marked limitation of physical activity. Comfortable at rest. Less than ordinary activity causes fatigue, palpitation, shortness of breath or chest pain
IV	Symptoms of heart failure at rest. Any physical activity causes further discomfort

Nonetheless, it should be noted here that in 1994 in order to increase the objectivity of the NYHA classifications, “objective assessment” classes (A–D) were added to the NYHA functional classes (I–IV). The objective assessment classes are determined on the basis of additional measurement tools, including electrocardiogram (EKG or ECG), echocardiogram (echo), radiologic images, stress tests, and x-rays. However, objective assessment is beyond the scope of this research, and thus will not be further considered.

Up to now, the heart failure (HF) classification has been the subject of the several studies. The majority of studies used the dataset from the UC Irivine Machine Learning Repository^18^, consisting of 303 datasets, of which 164 instances belong to the healthy class, whereas 139 instances belong to the heart disease class (from I to IV), each described by 14 clinical features, including the target attribute (see Table 2 for details). The summary of the identified studies, related to the heart disease classification, is given in Table 3.

**Table 2:** Dataset from the UC Irivine Machine Learning Repository.

Attributes	Type
Age	Real
Sex: Either male (0) or female (1)	Binary
Chest Pain type	Number
Resting blood pressure	Real
Serum cholestoral in mg/dl	Real
Fasting blood sugar>120 mg/dl	Binary
Resting electrocardiographic results (values 0,1,2)	Number
Maximum heart rate achieved	Real
Exercise induced angina	Binary
Oldpeak = ST depression induced by exercise relative to rest	Real
The slope of the peak exercise ST segment	Number
Number of major vessels (0-3) colored by flourosopy	Real
Thal: 3 = normal; 6 = fixed defect; 7 = reversable defect	Number
Class: Presence (1) and absence of heart disease	Binary

**Table 3:** Summary of various classification methods in terms of performance as measured by the F1-score.

Source	Method	Output classes	F1-score
Bashir et al.^8^	Naive Bayes	2	0.79
Bashir et al.^8^	Decision Tree	2	0.73
Bashir et al.^8^	Support Vector Machine	2	0.76
Bashir et al.^8^	Vote based Classifier	2	0.82
Uppin and Anusuya^35^	C4.5 Decision Tree	2	0.86
Tomar and Agarwal^32^	Feature selection based LSTSVM model	2	0.86
Aljaaf et al.^1^	C4.5 Decision Tree	5	0.86
Chaki et al.^12^	C4.5	2	0.7756
Chaki et al.^12^	Naive Bayes	2	0.835
Chaki et al.^12^	SVM	2	0.8412
Lee^21^	Rough-set-based rule classifier	2	0.84
Lee^21^	Neural network with weighted fuzzy membership functions without feature selection	2	0.856
Lee^21^	Neural network with weighted fuzzy membership functions with feature selection	2	0.874
Rjeily et al.^30^	CPT+	5	0.91
Liu et al.^25^	Ensemble classifier (*k* = 50)	2	0.8889
Liu et al.^25^	Ensemble classifier (*k* = 100)	2	0.9259
Liu et al.^25^	Ensemble classifier (*k* = 150)	2	0.9074
Liu et al.^25^	C4.5 tree	2	0.8703
Liu et al.^25^	Naive Bayes	2	0.8333
Liu et al.^25^	Bayesian Neural Networks	2	0.8519
Nashif et al.^28^	Naive Bayes	5	0.864
Nashif et al.^28^	SVM	5	0.9672
Nashif et al.^28^	Random Forest	5	0.9570
Nashif et al.^28^	Simple Logistic	5	0.95
Nashif et al.^28^	ANN	5	0.769
Li et al.^23^	Feature selection algorithm with Support Vector Machines	2	0.92

While conclusions drawn from samples are intended to be generalised to the population, some authors combined and unified different datasets into one. For example, Maambo et al.^27^ merged five datasets (Cleveland, Hungarian, Switzerland, Long Beach, and Stalog), resulting in a total of 1190 observations described by 12 features of which 11 are input classes and one is a dichotomous output class. This dataset is available for download on the Kaggle data platform^19^. In this case, the Bayesian data mining algorithm was used for class prediction, with an accuracy of 90.97 percent.

Obviously, there are more studies in which different datasets have been considered. For instance, Tripoliti et al.^34^ developed an automated method for the early identification of NYHA class change by using classification techniques. Based on a dataset of 378 instances, described by 102 features, HF severity was addressed as two, three and, for the first time, as four class classification problem, achieving detection accuracy of 97, 87 and 67 percent respectively.

Taking different lifestyles into account does not make the classification of heart failure a one-size-fits-all problem. So, there is room for more research that could look at different lifestyle factors. Having said that, in this study we will investigate a dataset that has only two attributes (age, diabetes) in common with those mentioned above, using the state-of-the-art classification methods.

## Methodology

In a general view, we followed the well-established and recognized guidelines^26^. Since the goal of the study is to build a prediction model for NYHA classification system, an obvious choice was to adopt a supervised learning approach, which means that the training dataset includes both observed inputs and correct outputs, allowing the model to learn over time^20^.

### Raw data

The dataset used in our analysis was collected by Professor Kinga Wegrzynowska-Teodorczyk from the 4. Wojskowy Szpital Kliniczny z Poliklinika in Wroclaw (Poland) between 2010 and 2019. The dataset includes information on 63 different variables for a total of 469 patients. These variables shed light on aspects such as patients’ fitness levels, the progression of their conditions, particularly heart failure, and other relevant clinical details. There were many challenges in studying this dataset. In particular, there were 4192 missing values scattered throughout the dataset. In addition to missing data, we also had to deal with issues such as proxies, outliers and multicollinearity between variables. To ensure the integrity and cleanliness of the data, we undertook several pre-processing steps to mitigate these challenges.

### Target attribute

The target of a supervised model is a special kind of attribute. The target column in the training data contains the historical values used to train the model. The target column in the test data contains the historical values to which the predictions are compared. The act of scoring produces a prediction for the target. In our analysis, the target attribute corresponds to the NYHA column. This column comprises integer values ranging from 1 to 4, representing the NYHA classification system for heart failure severity. However, it is worth noting the presence of proxy values, specifically 1.5, 2.5, and 3.5, as well as instances where the NYHA column may be missing a value (see Table 4). These factors necessitate the adaptation of appropriate preprocessing measures to address these anomalies effectively. Regarding the distribution of values within the target column, the shares are as follows:

**Table 4:** The data distribution of the NYHA values.

Value	Count	Share
1	77	16.739%
1.5	8	1.739%
2	222	48.261%
2.5	16	3.478%
3	125	27.174%
3.5	2	0.435%
4	10	2.174%

These shares represent the relative frequencies of occurrence of samples within each class. It is important to note that class sizes are not balanced, indicating an imbalance in the distribution of the target variable.

### Data preprocessing

Data preprocessing is a crucial step in the data mining process, the accuracy of the model is highly dependent on the quality of the data^15^. During the preprocessing phase, the dataset was cleaned and prepared for the analysis. The first step involved handling the NYHA column, where examples with proxy values and missing values were removed. The removal accounted for a total of 35 examples, leaving us with 434 examples to proceed with. Next, due to the large number of attributes, which amounted to 63, they were grouped into eight groups for better visualization and analysis. The groups included Clinical, Demographic, Technical, Anthropometry, Comorbidities, Treatment, Biochemistry, and Fitness level. With the attributes grouped, we proceeded to analyze each group separately. Our objective was to identify the missing values, outliers, and proxy values within the respective groups. By conducting individual analyses for each attribute group, we could gain a deeper understanding of their unique properties and address any data quality issues. In our study, we conducted an analysis on each group by removing the NYHA column, which served as the target attribute, and then examining the remaining attributes. Subsequently, we developed decision tree models based on the results of these analyses to determine the most influential attributes within each group. The process began with the removal of the NYHA column from each group, enabling us to focus solely on the attributes. We then applied analysis to investigate the relationships and dependencies among these attributes. By employing decision tree model, which is well-suited for classification tasks, we could discern the attributes that played a pivotal role in predicting the target NYHA classification. The primary objective of this analysis was to rank the attributes based on their correlation with the target attribute, thereby identifying the most influential features within each group. By selecting the top-ranking attributes, we selected the most important features to build the decision tree model specific to that particular group. This model served as a key component in one of the three approaches we employed, which was based on an ensemble learning method. Below are the descriptions and specific analyses conducted for each group.

#### Clinical group description and analysis

The clinical group consists of 12 attributes that offer pivotal clinical information relating to the health conditions of heart failure patients. Table 5 provides a comprehensive description of each attribute, highlighting its data type, the number of missing values, and a concise explanation of its relevance.

**Table 5:** Description of attributes in the clinical group.

Variable	Description	Data type	Missing values
DEATH?	Information if the patient is death (1), or alive (0)	Binary	77
QOL	Result of the survey measuring the quality of life (QoL, total score range 0–105, from best to worst)	Integer	183
OQLsub1	Scores for a QoL subscale—physical dimension (8 items, range 0–40 from best to worst)	Integer	206
OQLsub2	Scores for a QoL subscale—emotional dimension (5 items, range 0–25 from best to worst)	Integer	206
LVEF.0	Left ventricular ejection fraction—information from the heart ultrasound reflecting the efficiency of pumping. According to the definition value = 45 or lower is characteristic for systolic heart failure.	Integer	3
PM	Information about artificial pacemaker (0 = no pacemaker)	Binary	69
AETH.HF	Information about the clinical cause of heart failure (1 = ischemic disease or 2 = other)	Binary	4
LVEDD	Parameter from heart ultrasound: left ventricular end diastolic diameter (increased in heart failure)	Integer	133
MR	Mitral regurgitation (valvular heart disease), bigger number = worse	Real	138
REST.SBP	Systolic blood pressure at rest	Integer	105
REST.DBP	Diastolic blood pressure at rest	Integer	105
REST.HR	Heart rate at rest	Integer	106

The total number of missing values in the clinical group amounts to 1335. To ensure the data’s integrity, we implemented specific data handling techniques for each attribute.

For the attribute DEATH?, a binary variable indicating whether the patient has passed away or is still alive, all missing values were set to 0, signifying that the patients with missing values are presumed to be alive.

Regarding QOL an integer attribute presenting the result of a quality of life survey, with scores ranging from 0 to 105, some values exceeding the valid range. To rectify this, we capped all values larger than 105 to 105. For missing values, we employed backfill (bfill) method, which uses the next valid observation to fill the gap.

The attribute OQLsub1 which provides scores for a QoL subscale on focusing on the physical dimension (0-40) also contained values larger than 40. To address this issue, we capped all values larger than 40 to 40. Similar to previous attributes, missing values were imputed using the backfill method.

For OQLsub2, which represents scores for a QoL subscale on the emotional dimension (0–25), we found values exceeding the valid limit. Thus, we capped all values above 25 at 25. Again, the backfill method was employed for missing values.

For LVEF.0, an integer attribute that details the left ventricular ejection fraction from a heart ultrasound, only 3 missing values were noted. These were imputed using the backfill method. A total of 275 patients had an ejection fraction of less than 35%, which means that the heart pumps 35% or less of the blood out of the left ventricle (its main pumping chamber) with each beat. This is a severely impaired LVEF according to the British Society of Echocardiography’s recently updated normal reference intervals for assessing heart dimensions and function^17^.

For the binary attribute PM, which indicates whether a patient has an artificial pacemaker, all missing values were set to 0, suggesting that these patients do not possess an artificial pacemaker.

In the case of AETH.HF, a binary attribute pinpointing the clinical cause of heart failure, we found that values were consistently one unit higher than the defined categories. To correct this, 1 was subtracted from all values. Missing values were set to 0, indicating an undetermined cause for heart failure.

LVEDD, an integer attribute that stands for the left ventricular end diastolic diameter from a heart ultrasound, had 133 missing values. These were addressed using the backfill method.

For MR, a real number attribute detailing the severity of mitral regurgitation (valvular heart disease), the 138 missing values were replaced with the attribute’s most frequent value.

For the numerical attributes REST.SBP, REST.DBP, and REST.HR, which represent systolic blood pressure, diastolic blood pressure, and heart rate at rest, respectively, the backfill method was employed for missing value imputation.

**Figure 1:**
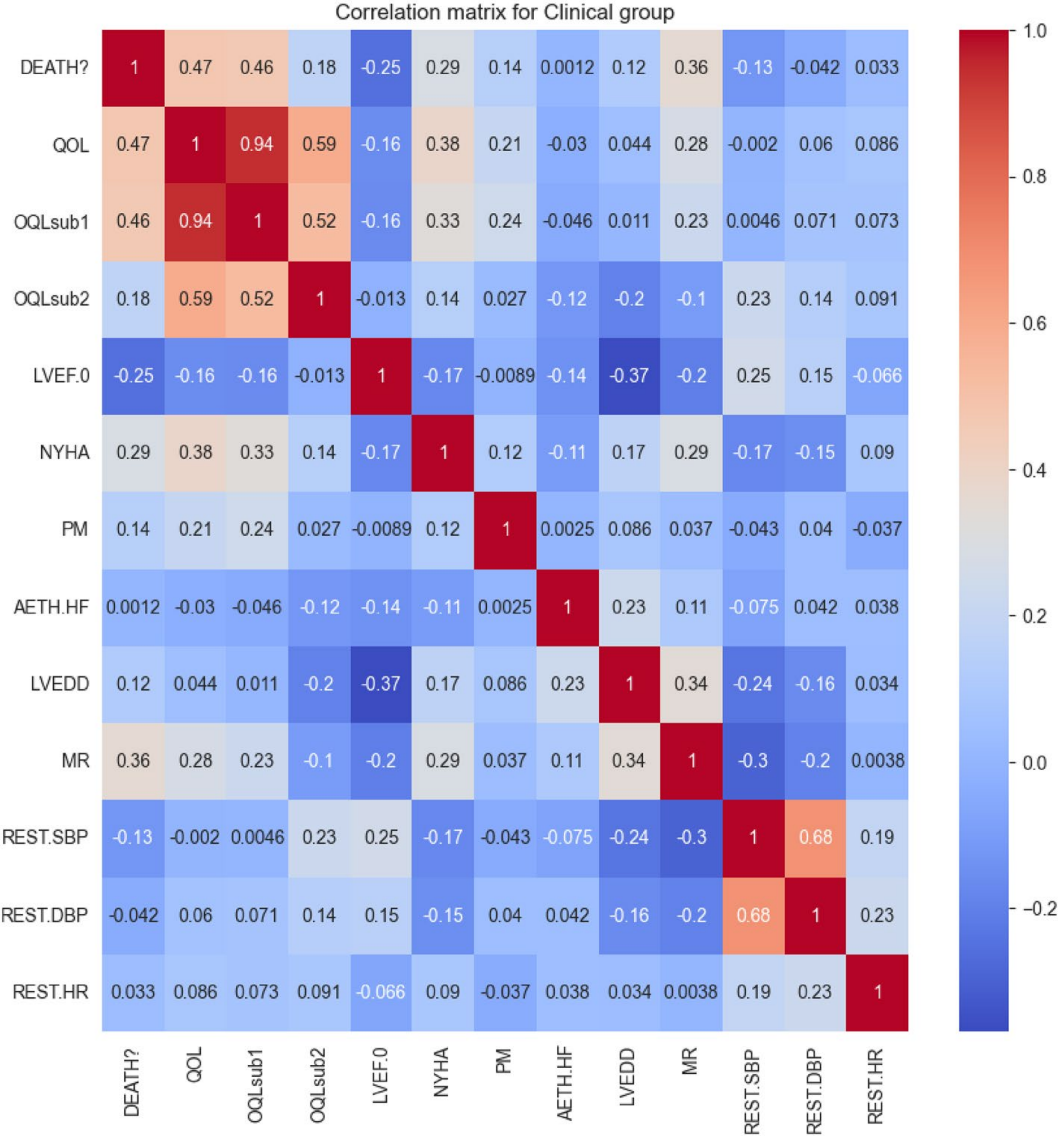
Heatmap of the correlation matrix for the Clinical Group.

Following these data handling approaches, we delved deeper into the analysis of the clinical group. A correlation matrix was computed, and its heatmap was plotted to understand inter-attribute relationships better. Figure 1 showcases the heatmap.

Each cell in the heatmap signifies the correlation coefficient between two attributes. The intensity of the cell’s color represents the strength of the correlation. By analyzing this, we unearthed critical insights into attribute relationships, which illuminated potential patterns and dependencies.

To enhance our model’s efficacy and curtail multicollinearity, attributes with a correlation coefficient below 0.18 to the target attribute were eliminated. This process streamlined our focus to the most pertinent features, reducing the attribute count from 12 to 4.

The attributes that were omitted include REST.SBP, LVEF.0, LVEDD, REST.DBP, OQLsub2, PM, AETH.HF, and REST.HR. OQLsub1 was also removed due to its high correlation with QoL, ensuring we minimized redundancy. Consequently, our analysis was narrowed down to 3 attributes: QoL, MR, and DEATH?, which were utilized to train our decision tree model and assess feature significance.

Subsequently, data preparation for the decision tree model ensued. The target attribute, NYHA, was distinguished from the feature set, marking the commencement of classifier training and attribute importance extraction.

Utilizing the decision tree classifier, we established a robust predictive model. Hyperparameter tuning was done using a grid search for optimal results. After selecting the best estimator from the grid search, our finalized clinical group model was derived.

Upon training the decision tree with the curated data, feature importances were extracted. This process allowed for the prioritization of attributes in predicting the target attribute. Table 6 enumerates these importances in descending order.

**Table 6:** Feature importances for the clinical group in descending order.

Variable	Importance
QOL	0.813847
MR	0.138863
DEATH?	0.04729

In conclusion, the feature importances identified the most pertinent attributes within the clinical group. The QOL attribute emerged as the most influential, followed by MR and then DEATH?. These attributes will be central in our subsequent analyses.

#### Demographic group description and analysis

The demographic group in our study focuses on a single variable AGE. This variable represents the age of individuals at the time of examination, and its serves as a crucial factor in understanding how age influences heart failure patients’ functional status.

Before proceeding with the analysis, we validated the integrity of the AGE data. We ensured that the AGE column contained integer values and checked for any missing data. To address missing values, we utilized a backfill method to impute the data. Additionally, since age is typically represented as a integer number, we rounded the age values from decimal to integer.

**Figure 2:**
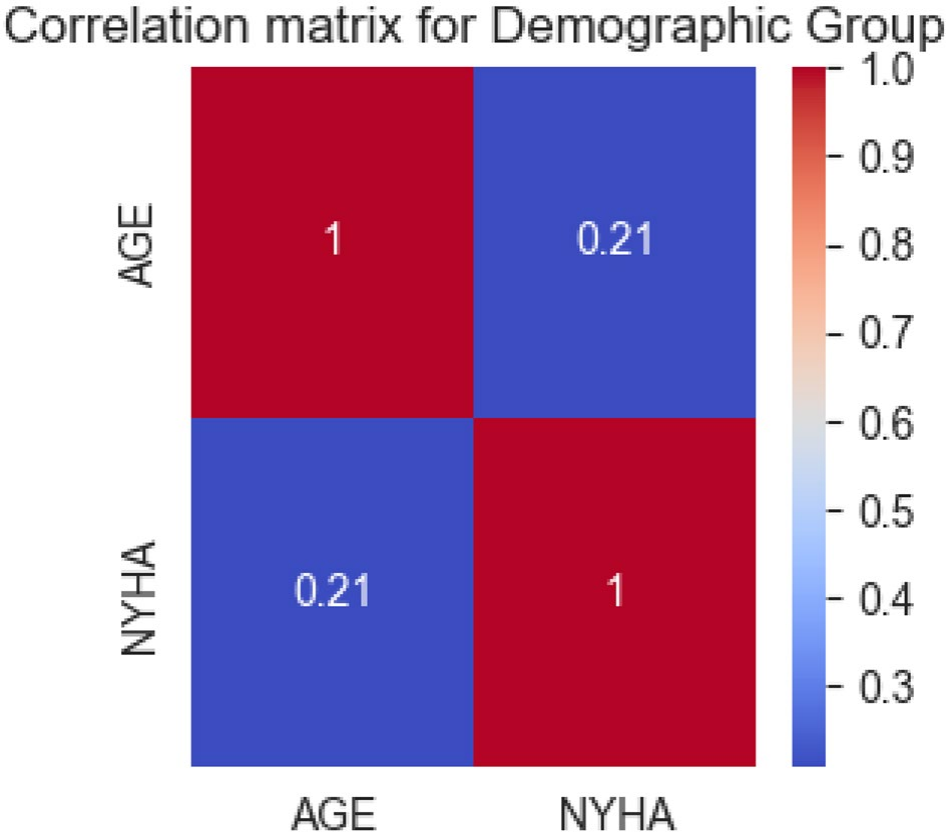
Heatmap of the correlation matrix for the Demographic Group.

To explore the relationship between age and the NYHA classification, we visualized the correlation between these two variables. Figure 2 presents the correlation between age and NYHA classification.

The correlation between age and NYHA classification was 0.21 indicating a medium positive correlation between age and NYHA classification. As age increases, the NYHA classification also increases. This result is consistent with our hypothesis that age is a significant factor in determining the NYHA classification.

While we do not develop a predictive model for the demographic group due to presence of a single variable, our analysis of age and its impact on NYHA provides valuable insights will be used in the our overall predictive model.

#### Technical group description and analysis

The technical group comprises variables that are not directly related to the patient’s health but provide essential supplementary information. Table 7 presents the variables in the technical group.

**Table 7:** Description of attributes in the technical group.

Variable	Description	Data type	Missing values
DEATHDATE	Date of death (if death = 1) or date of the confirmation that the patient is still alive	Date	78
TIMEFU	Number of days between examination and date death or date of the confirmation that the patient is still alive	Integer	97
DOB	Date of birth	Date	7
DOE	Date of the examination	Date	0

During the analysis of the technical group, we encountered 182 missing values. To address this issue and ensure data quality, we used specific data handling techniques for each attribute. For the attributes DEATHDATE, TIMEFU and DOB, we used the backfill method for data imputation.

The DOE attribute, indicating the date of the examination, did not contain any missing values. Therefore, no further handling was required for this attribute.

**Figure 3:**
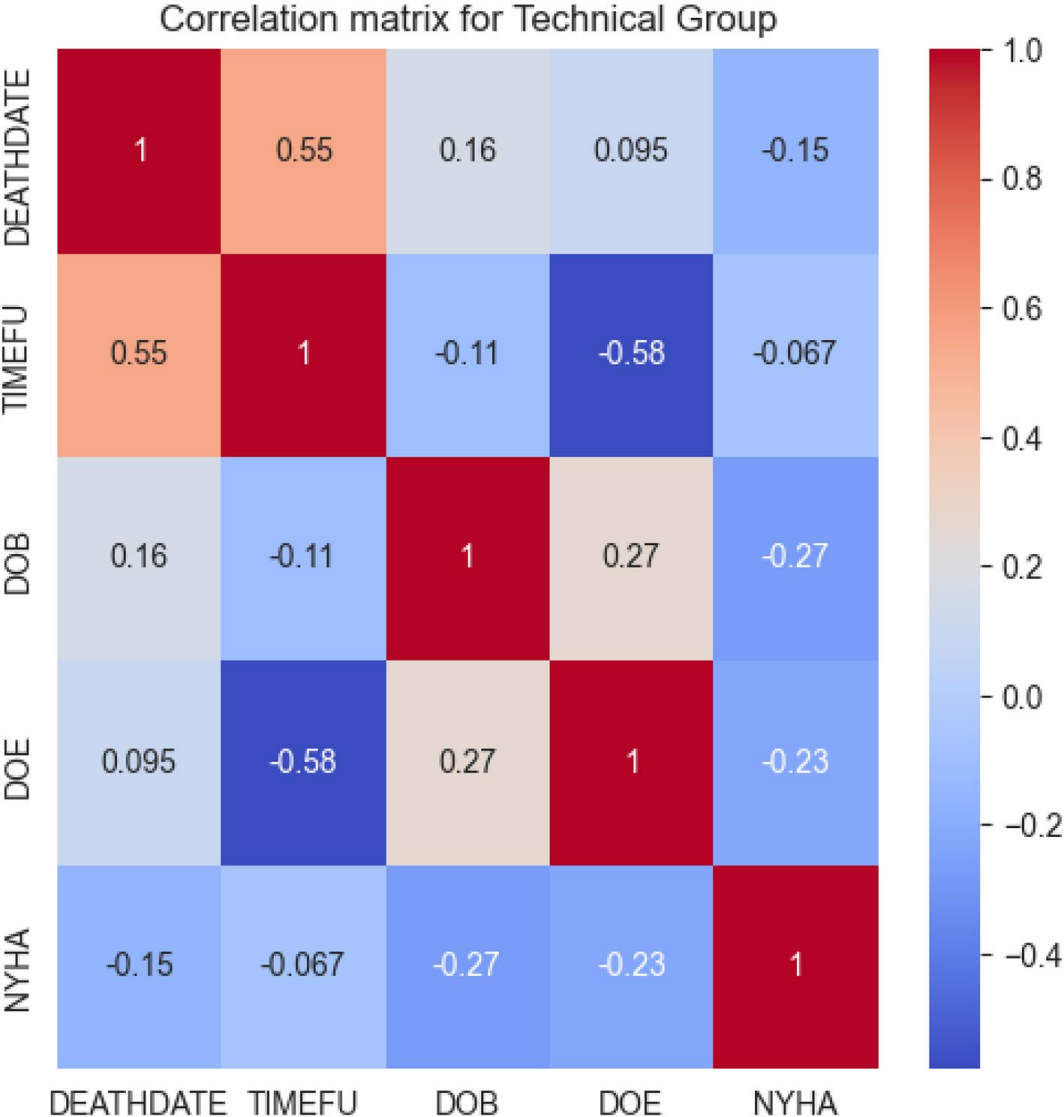
Correlation matrix for the technical group.

To better understand the relationships among the attributes in the technical group, we conducted a correlation analysis. Figure 3 presents the correlation matrix for the technical group.

However, the correlation matrix did not provide any meaningful insights. Consequently, we concluded that the variables within the technical group do not contribute significantly to predicting the NYHA functional classification in heart failure patients. Because of this, and the fact that the attributes DOB and DOE are related with the AGE attribute from the demographic group, we decided to exclude the technical group from our analysis.

#### Anthropometric group description and analysis

The antropometric group refers to measurements that determine the size, shape, and composition of the human body. Whlie they might not offer direct insights into a patient’s health, they provide context that can influence clinical desisions and understanding of health trajectories. Table 8 provides an overview of the variables in the anthropometric group.

**Table 8:** Description of attributes in the anthropometric group.

Variable	Description	Data type	Missing values
HEIGHT	Patient’s height in centimeters	Real	0
WEIGHT	Patient’s weight in kilograms	Real	0
BMI	Body mass index, calculated as weight divided by height squared	Real	0

The anthropometric group did not contain any missing values. Therefore, no further handling was required for this group.

**Figure 4:**
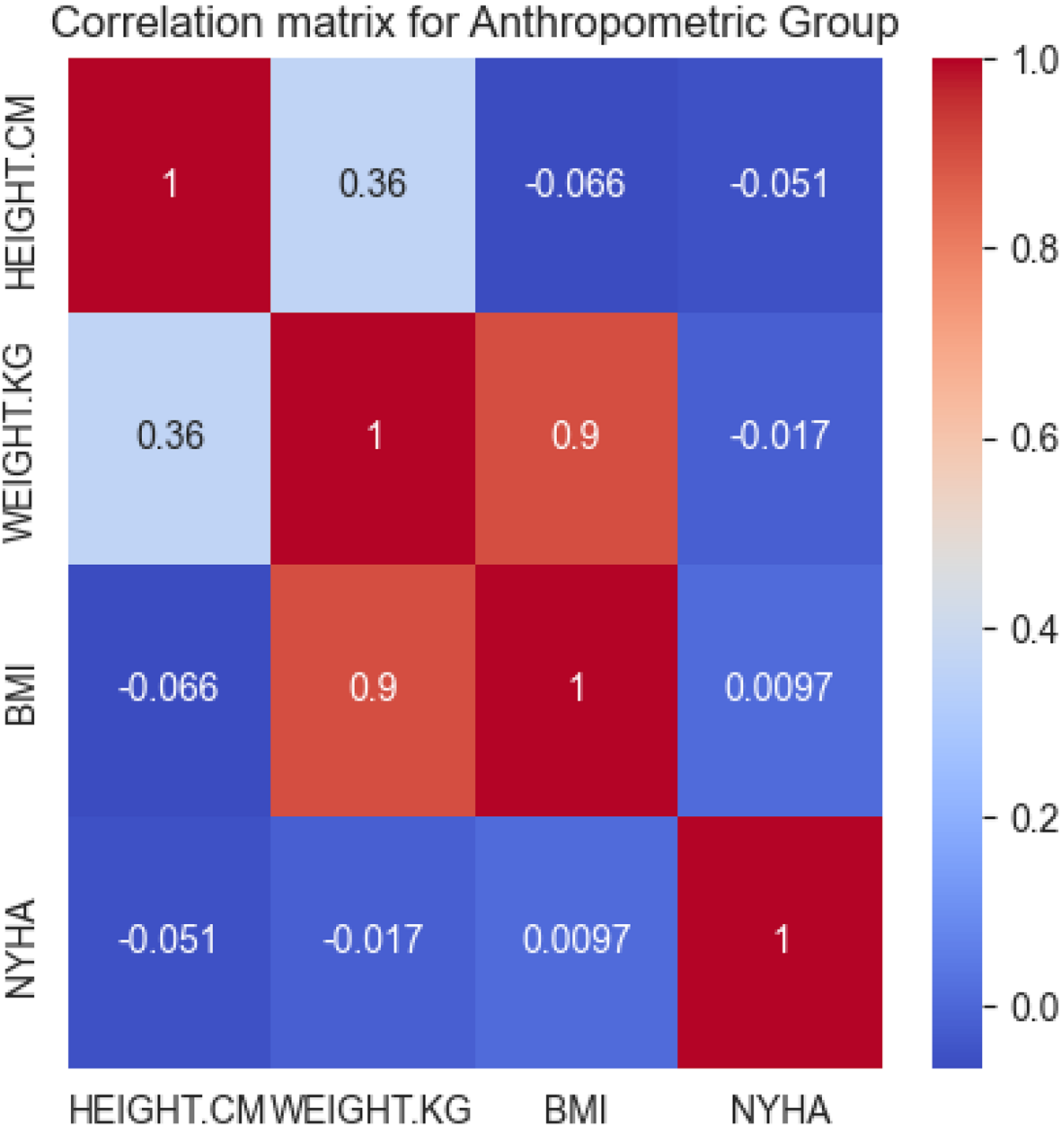
Correlation matrix for the anthropometric group.

To discern the relationships among the attributes in the anthropometric group and their connection with the NYHA functional classification, we conducted a correlation analysis. The results are presented in Fig. 4.

From the correlation matrix, it is evident that the correlation between the antropometric attributes (HEIGHT, WEIGHT, and BMI) and the NYHA functional classification is minimal. The highest correlation is between the BMI and WEIGHT (0.9), which is expected as BMI is derived measure from the HEIGHT and WEIGHT. However, their correlation with the NYHA class is extremely low (− 0.017 for WEIGHT, and 0.0097 for BMI).

In light of these findings, and given the lack of significant correlation with the NYHA class, we have decided to exlude the anthropometric group from our further analysis for predicting the NYHA functional classification in heart failure patients. However, it is important to note that obesity, as measured by body mass index (BMI), is commonly associated with increased cardiovascular risk and heart failure^16,36^. Therefore, these results should be treated with caution.

#### Comorbidities group description and analysis

The comorbidities group encompasses variables that denote the presence of other coexisting chronic diseases or conditions in the patients. These comorbidities may significantly affect the course and prognosis of heart failure. Table 9 summarizes the variables in this group.

**Table 9:** Description of attributes in the comorbidities group.

Variable	Description	Data type	Missing values
MI	Information about previous myocardial infarction (1 = yes)	Binary	21
AF	Information about atrial fibrillation (1 = yes)	Binary	4
DM	Information about diabetes (1 = yes)	Binary	21
HT	Information about hypertension (1 = yes)	Binary	95
COPD	Information about lung disease (1 = yes)	Binary	37
STROKE	Information about previous stroke (1 = yes)	Binary	35
KIDNEY.DIS	Information about kidney disease (1 = yes)	Binary	70

There were 283 missing values in total. Each missing values for the comorbidities group was imputed with the 0, indicating the absence of the corresponding comorbidity.

**Figure 5:**
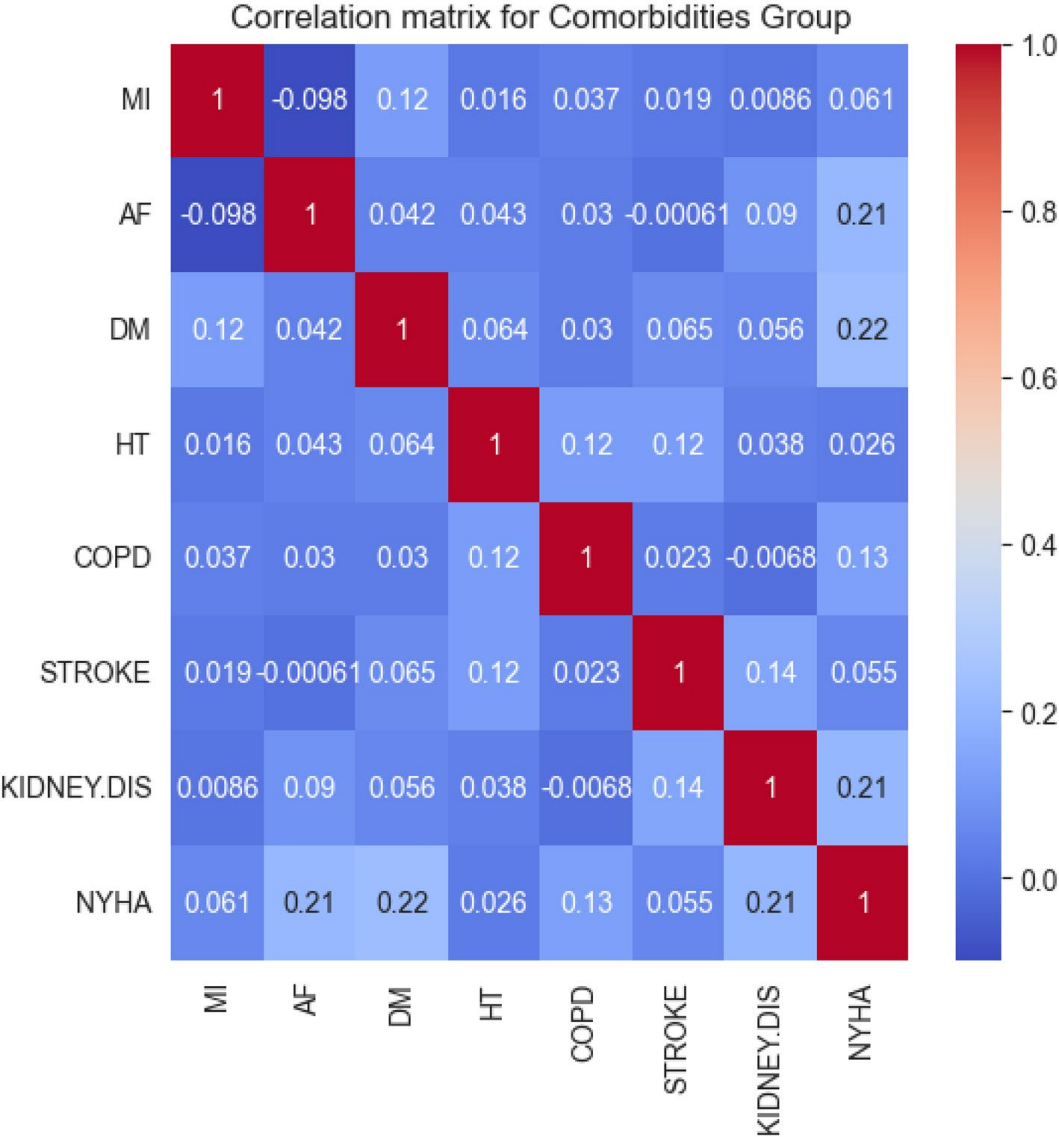
Correlation matrix for the comorbidities group.

The correlation matrix for this group (presented in Fig. 5) suggests a relatively stronger correlation between certain comorbidities and the NYHA class compared to the previous groups.

The DM (diabetes mellitus), AF (artial fibrillation), and KIDNEY.DIS (kidney disease) variables exhibit the strongest correlation with the NYHA class, each with correlations of 0.22, 0.21, and 0.21 respectively. Additionaly, COPD (chronic obstructive pulmonary disease), despite having a lower correlation, has a correlation of 0.13. HT (hypertension) has a relatively lower correlation of 0.026, while MI (myocardial infarction) and STROKE have correlations 0f 0.061 and 0.055 respectively. These correlations suggest that these comorbidities may influence the severity of heart failure, as classified by the NYHA.

Following the exclusion of attributes with correlation lower than 0.15, we retained DM, AF, and KIDNEY.DIS for further analysis.

By focusing on these specific comorbidities, we derived an optimized predictive model. This model, based on the selected features, aims to predict the NYHA classification efficiently. This approach allows us to uncover the most substantial comorbidities that affect the severity of heart failure. In the following table (Table 10), we present the final list of the most influential comorbidities, in descending order of importance.

**Table 10:** Feature importances for the comorbidities group in descending order.

Variable	Importance
DM	0.442883
KIDNEY.DIS	0.342886
AF	0.214231

The results suggest that diabetes mellitus is the most influential comorbidity, followed by kidney disease and atrial fibrillation. These comorbidities were found to be the most critical contributors to the NYHA based on their strong correlations and feature importances, thus we have decided to retain them for further analysis, and include them as representative comorbidities for the comorbidities group.

#### Treatment group description and analysis

The treatment group is composed of 5 attributes, each detailing a specific treatment regimen for heart failure patients. A detailed description of these attributes is provided in Table 11.

**Table 11:** Description of attributes in the treatment group.

Variable	Description	Data type	Missing values
ACEI.ARB	Information about treatment using ACE inhibitors or ARBs (similar drugs, 1 = yes, 0 = no)	Binary	1
BB	Information about treatment using beta blockers (1 = yes, 0 = no)	Binary	1
MRA	Information about treatment using aldosterone antagonists (1 = yes, 0 = no)	Binary	12
DIUR	Information about treatment using oral diuretics (1 = yes, 0 = no)	Binary	5
ANTIPLAT	Information about treatment using antiplatelet drugs (1 = yes, 0 = no)	Binary	1
STATIN	Information about treatment using statin (1 = yes, 0 = no)	Binary	2
DIGOX	Information about treatment using digoxin (1 = yes, 0 = no)	Binary	2

The total number of missing values in the treatment group is 24. To address this issue, for each binary attribute indicating the medication the patient is taking, we set all missing values to 0, signifying that the patients with missing values are not taking the corresponding medication.

**Figure 6:**
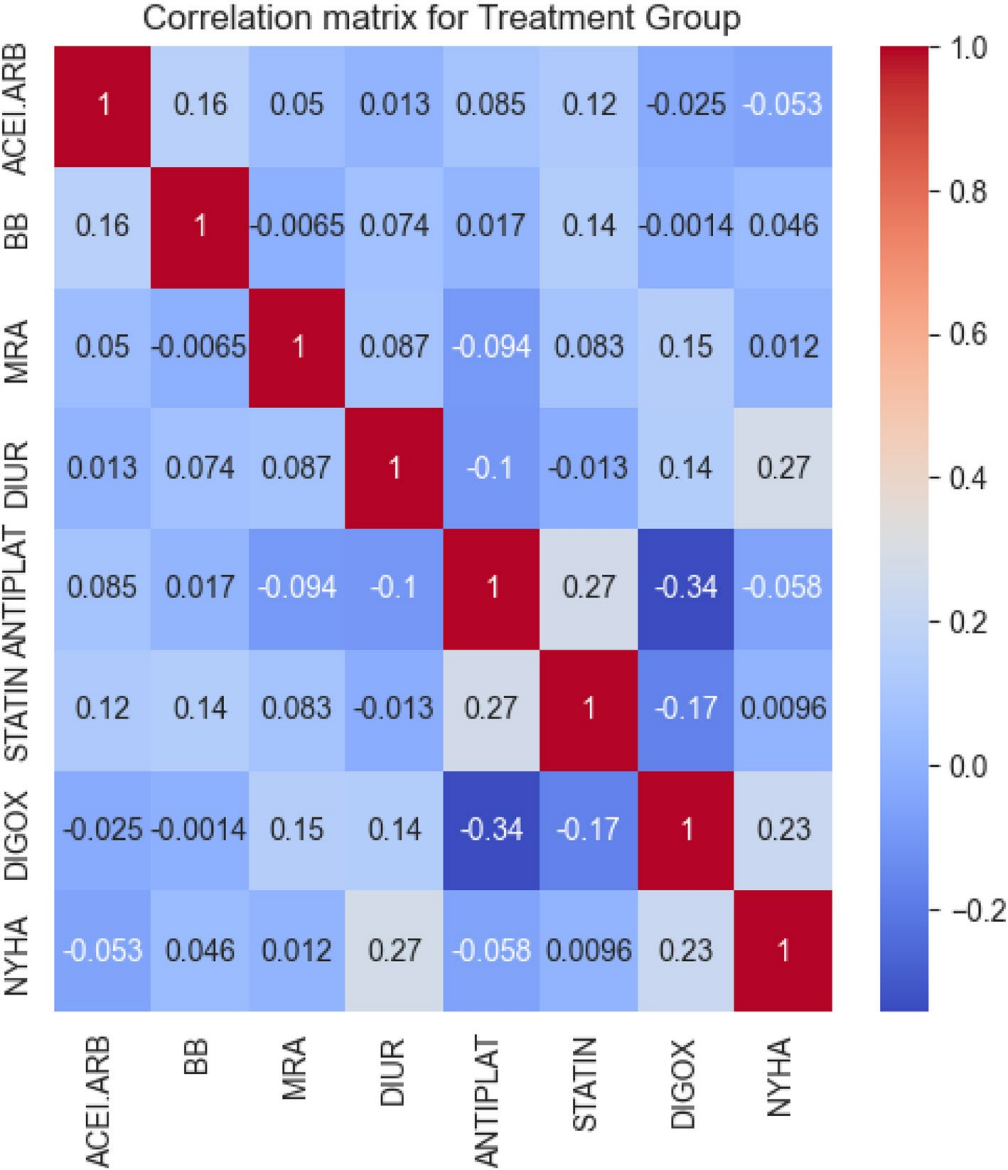
Heatmap of the correlation matrix for the Treatment Group.

Upon this preliminary data handling, we further scrutinized the relationships among the treatment attributes. We computed a correlation matrix and visualized it in a heatmap as shown in Figure 6.

Using a correlation threshold of 0.2, we honed our focus on the treatments showing substantial correlations with the NYHA classification. Specifically, DIGOX (digoxin treatment) and DIUR (oral diuretic treatment) demonstrated correlations of 0.23 and 0.27, respectively, with NYHA scores.

This suggests that these treatments are notably associated with advanced heart failure stages, as indicated by elevated NYHA scores. Accordingly, we used only the DIGOX and DIUR attributes to train the decision tree model, the importances of which are displayed in Table 12.

**Table 12:** Feature importances for the treatment group in descending order.

Variable	Importance
DIUR	0.663674
DIGOX	0.336326

This model presented feature importances of 0.66 and 0.34 for DIUR and DIGOX, respectively. These importances indicates that the DIUR carries more weight in the model’s determination of the NYHA classification, asserting its significance within the treatment group. In conclusion, DIGOX and DIUR are both selected as the final representative attributes of the treatment group due to their discernible impact on heart failure.

#### Biochemistry group description and analysis

The biochemistry group consists of variables that depict the results of biochemistry tests, thus providing insights into the physiological state of the patients with heart failure. Table 13 below gives a detailed description of the attributes in the biochemistry group.

**Table 13:** Description of attributes in the biochemistry group.

Variable	Description	Data type	Missing values
HB	Level of blood hemoglobin	Real	12
NA	Level of blood sodium	Real	11
K	Level of blood potassium	Real	12
BNP	Level of blood peptide BNP (Elevated level is characteristic for heart failure)	Real	41
CRP	Level of blood protein CRP (Characteristic for inflammation)	Real	126

The biochemistry group contains a total of 202 missing values distributed among its attributes. In handling these, the bfill method was applied to each attribute. This strategy uses the next valid observation in the dataset to fill the gap caused by a missing value.

**Figure 7:**
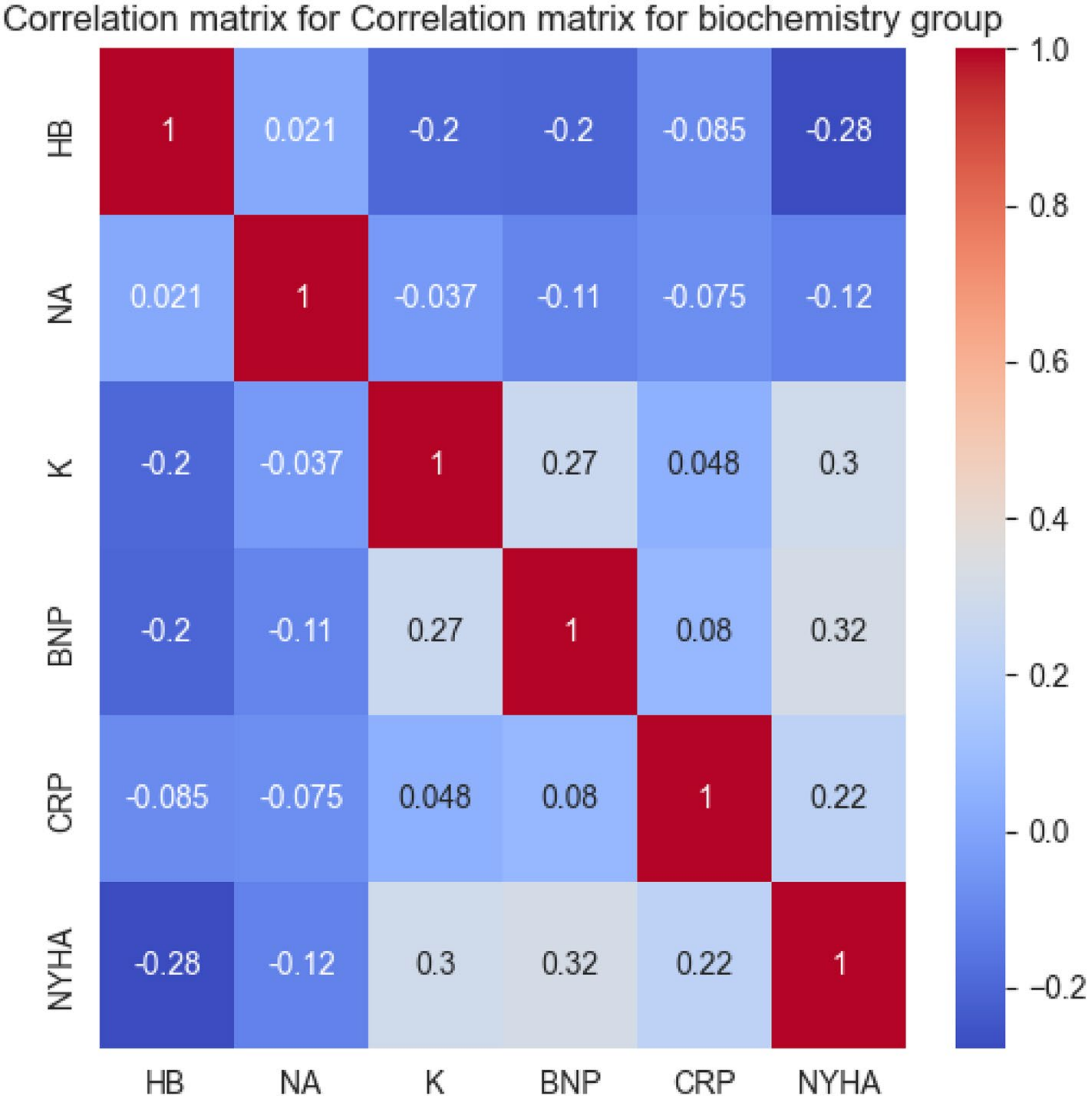
Heatmap of the correlation matrix for the biochemistry group.

To investigate the relationships among the variables in the Biochemistry group, a correlation matrix was computed and visualized in a heatmap as shown in Fig. 7.

Setting a correlation threshold of 0.25, a clearer understanding of the relationships between the biochemistry variables and NYHA classification emerged. The variables BNP, K, and HB were found to exhibit statistically significant correlations with the NYHA, with respective correlation coefficients of 0.32, 0.3, and − 0.28.

These correlations suggests that the levels of BNP, K, and HB may have notable associations with the NYHA classification. Consequently, subsequent iterations of the decision tree model were trained exclusively using the BNP, K, and HB attributes. The Table 14 below ilustrates the feature importance for the Biochemistry group in descending order.

**Table 14:** Feature importances for the biochemistry group in descending order.

Variable	Importance
BNP	0.393258
K	0.339789
HB	0.266953

The decision tree model computed feature importances of 0.393258, 0.339789, and 0.266953 for BNP, K, and HB, respectively. These values suggest that within the Biochemistry group, the BNP, K, and HB variables have a significant influence on the model’s prediction of NYHA classification. Therefore, BNP, K and HB are selected as the final representative features of the Biochemistry group.

#### Fitness level group description and analysis

The Fitness Level Group consists of variables that provide insights into a patient’s fitness level. These variables range from simple exercise tasks to more detailed metrics derived from treadmill tests. The Table 15 below gives a detailed description of the attributes in the Fitness Level Group.

**Table 15:** Description of attributes in the fitness level group.

Variable	Description	Data type	Missing values
EXERCISE1	Number of seconds needed to complete the task (higher = worse)	Real	6
EXERCISE2	Number of repeated movements during the task (higher = better)	Real	6
EXERCISE3	Number of repeated movements during the task (higher = better)	Real	6
6MWT.DIST	Distance covered by the patient during the 6MWT (higher = better)	Real	5
6MWT.FATIGUE	Level of fatigue after 6MWT, scale 0–10	Integer	88
6MWT.DYSPN	Level of breathlessness during 6MWT, scale 0–10	Integer	88
6MWT.SBP1	Systolic blood pressure before the 6MWT	Real	47
6MWT.DBP1	Diastolic blood pressure before the 6MWT	Real	48
6MWT.HR1	Heart rate before the 6MWT.	Real	48
6MWT.SBP2	Systolic blood pressure after the 6MWT	Real	48
6MWT.DBP2	Diastolic blood pressure after the 6MWT	Real	48
6MWT.HR2	Heart rate after the 6MWT	Real	50
EXERCISE4	Patient’s ability to touch feet with fingers of palms (higher = better)	Real	68
EXERCISE5	Patient’s ability to touch left palm with the right one at back (higher = better)	Real	69
CPX.TIME	Time of exercise on a treadmill	Real	114
CPX.PEAKVO2	Peak oxygen consumption during treadmill exercise	Real	187
CPX.PEAKVO2FORBM	Peak oxygen consumption per body mass during treadmill exercise	Real	51
RER	Respiratory exchange ratio during treadmill exercise	Real	81
SLOPE	Slope between oxygen and carbon dioxide during treadmill testing	Real	52
METS	Number of metabolic equivalents during treadmill exercise	Real	182
WEBER	Weber classification for physical capacity (higher class = worse)	Integer	51
PEAK>18	Patients divided by peak oxygen consumption cutoff	Binary	51
SLOPE>35	Patients divided by slope cutoff for Weber classification	Binary	85

In this group, we identified a total of 1479 missing values distributed across various attributes. To address these, we implemented a range of strategies tailored to the extent of the missing data. For attributes with a relatively small number of missing values, such as EXERCISE1, EXERCISE2, EXERCISE3, and 6MWT.DIST, we replaced the gaps with the mean of the respective attribute.

For attributes that had a larger volume of missing data, like 6MWT.FATIGUE, 6MWT.DYSPN, CPX.TIME, and CPX.PEAKVO2, we adopted a more sophisticated backfill method. This approach ensures that these columns are both accurate and complete.

It’s important to note that attributes such as 6MWT.FATIGUE, 6MWT.DYSPN, and CPX.PEAKVO2 had a considerable number of missing values. Despite this challenge, we opted to retain them in our dataset, leveraging the backfill strategy to ensure their integrity.

After these measures, we reviewed the dataset and confirmed that all missing values had been successfully addressed, laying a solid foundation for the subsequent analysis.

**Figure 8:**
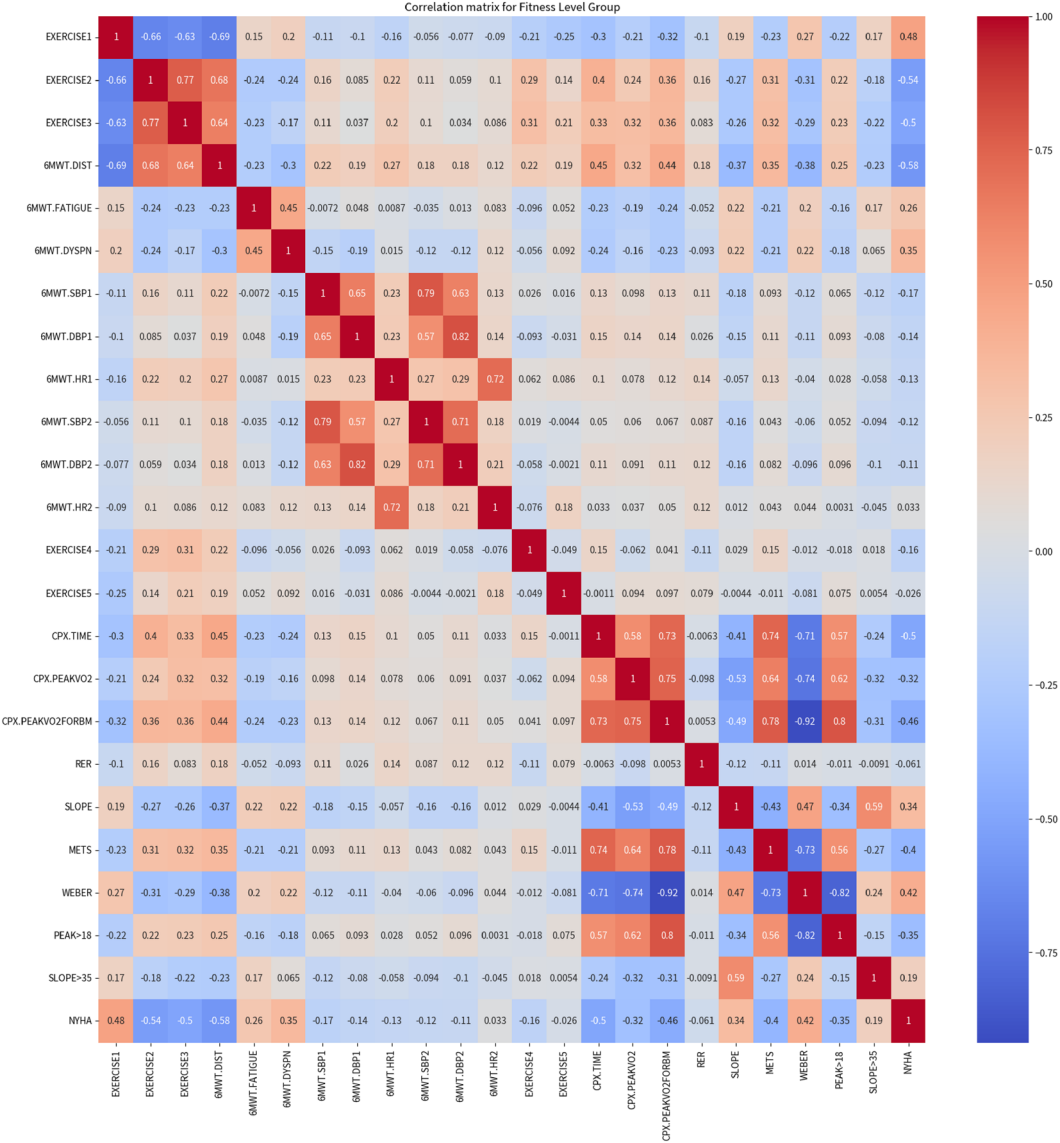
Heatmap of the correlation matrix for the Fitness Level Group.

For better insights into the Fitness Level Group, we computed the correlation matrix for each attribute in the group. The heatmap in figure 8 below illustrates the correlation matrix for the Fitness Level Group.

In our analysis of the correlation matrix, we set a threshold of 0.35 for correlation with the target attribute. Based on this threshold, we identified the following 10 attributes as having a strong correlation with the target attribute: 6MWT.DIST, EXERCISE2, EXERCISE3, CPX.TIME, EXERCISE1, CPX.PEAKVO2FORBM, WEBER, METS, PEAK>18, 6MWT.DYSPN.

However, upon a closer examination of inter-variable relationships, we observed a high degree of correlation between amoing these attributes. Such multicollinearity can be problematic, particulary when developing models like decision tree, as it can result in overfitting and reduced interpretability.

Considering this, for the construction of our decision tree, we opted to use only the 6MWT.DIST, CPX.TIME, and EXERCISE1 attributes. These attributes had the highest correlation with the target attribute, while also having a low degree of correlation with each other.

In the Table 16 below, we present the feature importances in descending order for the chosen representative attributes.

**Table 16:** Feature importances for the fitness level group.

Attribute	Importance
6MWT.DIST	0.519437
CPX.TIME	0.377920
EXERCISE1	0.102643

By focusing on these pivotal attributes, we decided to exclude the remaining attributes in the Fitness Level Group from our analysis. This approach allowed us to reduce the complexity of our model, while also ensuring that the most important attributes were included.

### Methods

In the conducted research we used three models of learning, including the CART algorithm^11^, and two ensemble methods, viz: Voting Classifier^5^ and Random Forest^4^, available in the scikit-learn version 1.4.2.

#### Method using CART algorithm

CART, which stands for Classification And Regression Trees, is a straightforward technique used for both classification and regression tasks. It operates by building a binary tree where every node signifies a specific check or condition on a single attribute. The tree grows from the top-down, employing a recursive method that continually divides data until it meets certain criteria. The process stops either when all instances at a particular node fall into a single category or when further division doesn’t enhance the predictive accuracy. In our study we took advantage of the CART algorithm from Scikit-Learn library. For the Decision Tree Classifier, we employed the Gini criterion which measures the frequency at which a randomly chosen element would be incorrectly classified. The tree was constrained to a maximum depth of 6 levels (max_depth = 6), ensuring simplicity and interpretability. We also specified that each leaf node should contain at least 4 samples (min_samples_leaf = 4), and any internal node requires at least 4 samples to consider a further split (min_samples_split = 4). All other hyperparameters of the model were set to their default values.

**Figure 9:**
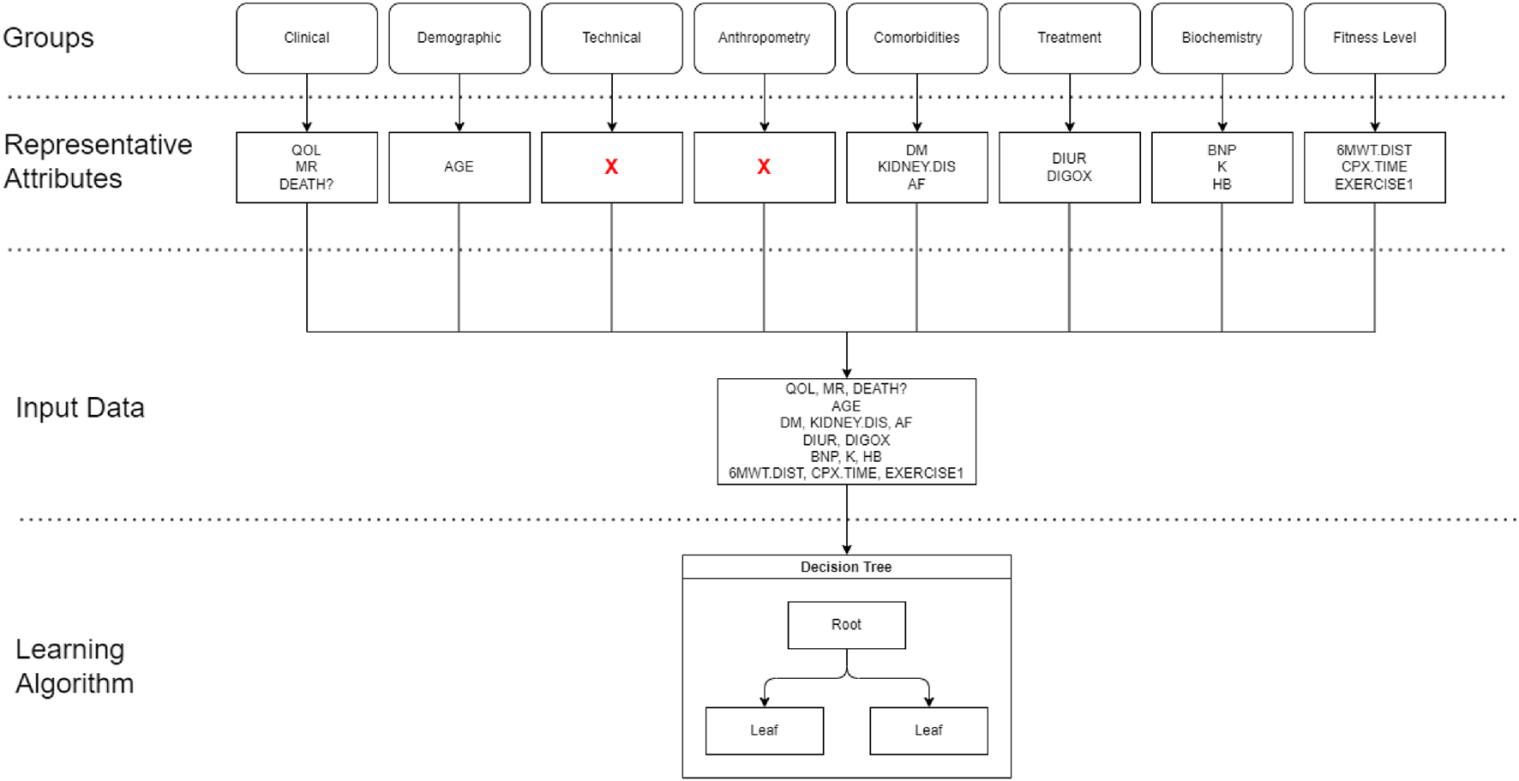
Flow chart of data processing for Cart algorithm.

The data preprocessing phase enabled us to isolate a subset of key attributes from each group, which were then employed to construct the decision tree. The procedural breakdown of the data processing is depicted in the flow chart shown in Fig. 9 below.

From the diagram, it’s evident which attributes were deemed essential from each group, following the preprocessing steps. Our dataset comprises 434 instances and 15 pivotal attributes (presented in Table 17), which served as the foundation for the construction of the decision tree.

**Table 17:** Summary of the selected attributes for the classification model.

Variable	Data type & description
OQL	Integer; Result of the survey measuring the quality of life (QoL) of the patient, with range from 0 to 105 (*from the best to the worst*)
MR	Real; The mitral regurgitation (valvular heart disease) (*from the best to the worst*)
DEATH?	Binary; information if the patient is death (1), or alive (0)
AGE	Integer; patient age at the time of examination
DM	Binary; information about diabetes (1 = yes)
KIDNEY.DIS	Binary; information about kidney disease (1 = yes)
AF	Binary; information about atrial fibrillation (1 = yes)
DIUR	Binary; information about treatment using oral diuretics (1=yes)
DIGOX	Binary; information about treatment using digoxin (1 = yes)
BNP	Real; Level of blood peptide BNP (Elevated level is characteristic for heart failure)
K	Real; Level of blood potassium.
HB	Real; Level of blood hemoglobin.
6MWT.DIST	Integer; the distance (in meters) covered by the patient during the 6 minute walking test (*the higher the value the better*)
CPX.TIME	Real; the exercise duration (in minutes) on a treadmill (*the higher the value the better*)
EXERCISE1	Real; the duration (in minutes) needed to complete the task (*the higher the value the worse*)

#### Method using voting classifier

The Voting Classifier represents an ensemble learning technique. Its core principle is to combine various distinct machine learning classifiers and use either a majority consensus or the mean of predicted probabilities (soft voting, where voting parameter is set to “soft”) to derive the class labels. Employing such a classifier is advantageous when working with a set of models that perform equivalently well, helping to counterbalance their individual shortcomings. In our study, we utilized the soft voting mechanism of the Voting Classifier algorithm from the Scikit-Learn library. Through this soft voting method, the class label is determined by choosing the one with the highest aggregated predicted probability. All other hyperparameters of the model were set to their default values.

**Figure 10:**
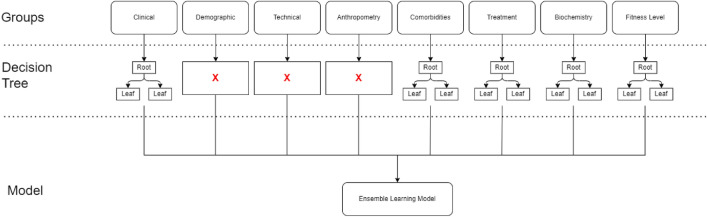
Overview of data preprocessing for the voting classifier.

For data preprocessing in the groups of Clinical, Comorbidities, Treatment, Biochemistry, and Fitness Level, we established straightforward decision tree models for each specified group. The procedure followed during this data preprocessing is visualized in the flow chart in Fig. 10.

The chart clearly showcases the creation of decision tree models tailored for each group. Subsequently, utilizing the Voting Classifier approach, we amalgamated these models to formulate the final comprehensive model. This finalized model was trained on the identical dataset that the decision tree model, developed through the CART algorithm, was trained on.

#### Method using Random Forest

Random Forest, a ensemble machine learning technique, establishes its foundation on a multitude of decision trees during the training phase. In the Random Forest algorithm, these individual trees are constructed based on random subsets of the data through a process called bootstrapping. Additionally, at each split, a random subset of the features is considered. This randomness ensures diversity among the trees and reduces the potential of overfitting, making the ensemble more robust compared to a single decision tree.

For our study, we employed the Random Forest algorithm from the Scikit-Learn library. Our model is composed of 100 individual decision trees

(n_estimators = 100). Furthermore, we’ve set a constraint ensuring that each tree doesn’t exceed a depth of 10 (max_depth=10), striking an optimal balance between model complexity and performance. The dataset used for the Random Forest model was consistent with the one utilized for earlier techniques. All other parameters were left at their default values.

### Ethical approval

All methods were carried out in accordance with relevant guidelines and regulations. Informed consent was obtained from all individual participants included in the study. Informed consent to participate in the study was obtained from all participants. Experimental protocols was approved by the Ethics Committee of Wojskowy Szpital Kliniczny z Poliklinka in Wroclaw.

## Results

To evaluate our models, we employed a 10-fold cross-validation method, incorporating stratification for testing purposes^13^. This cross-validation process was undertaken ten times, each time with unique random splits of the dataset into training and test subsets^22^. We used stratification to guarantee that every fold retained a consistent distribution of the target variable^33^. The model’s accuracy was measured by averaging the accuracy over all ten iterations of cross-validation^6^.

To further understand our model’s effectiveness, we analyzed its confusion matrix, which is a tool that presents a comprehensive view of the model’s accuracy by comparing its predictions against actual outcomes. This matrix segregates outcomes into four categories: true positives (TP), true negatives (TN), false positives (FP), and false negatives (FN). The true positive and true negative values denote accurate predictions, while false positive and false negative values indicate errors.

From the confusion matrix, we extracted key performance indicators, such as precision, recall, and the F1 score. Precision captures the fraction of accurate predictions within a class relative to all predictions for that class. Recall quantifies the fraction of accurate predictions within a class relative to all actual instances of that class. The F1 score, on the other hand, is the harmonic average of precision and recall, serving as a balanced metric. An F1 score closer to 1 indicates a more effective classification.

### Decision Tree model

**Figure 11:**
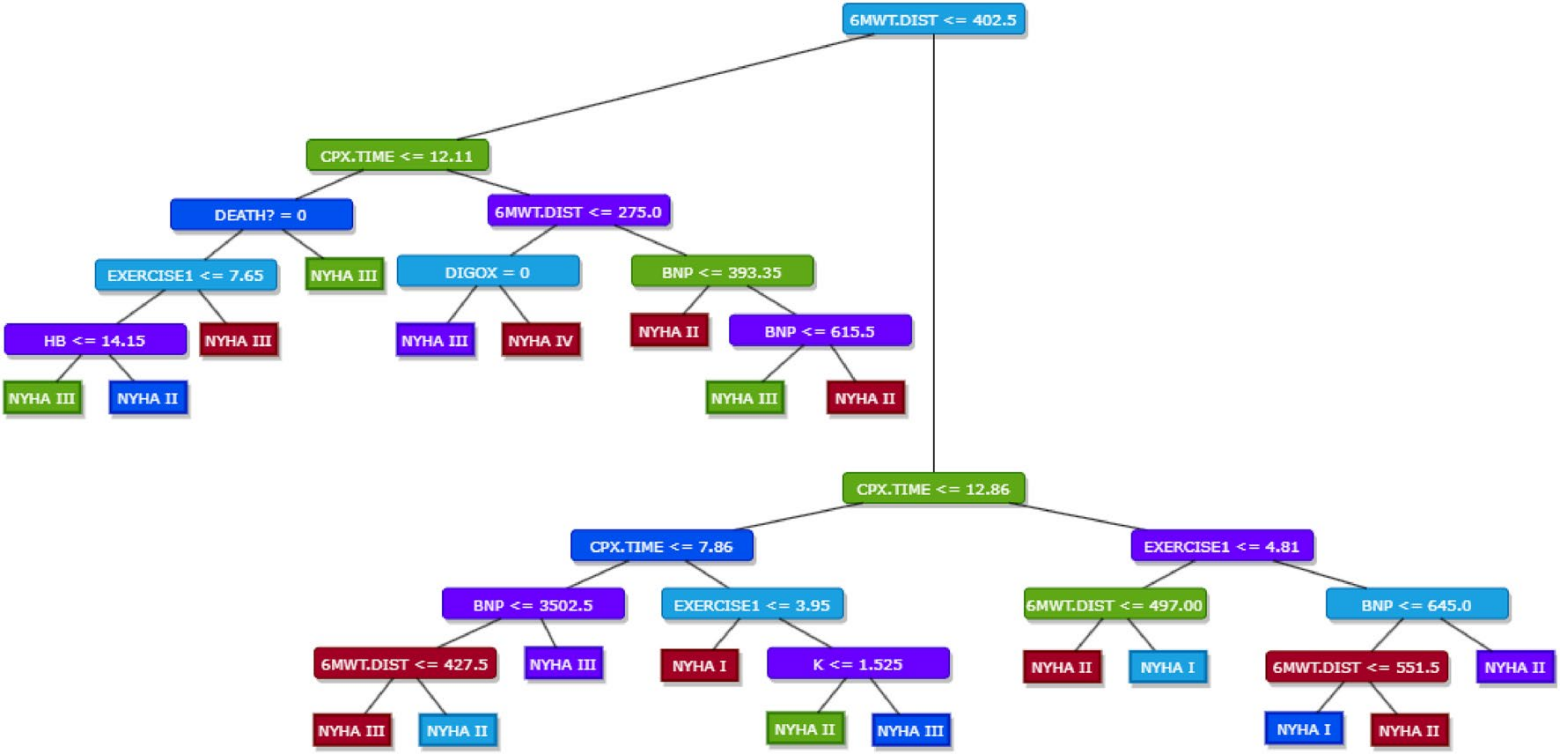
Decision Tree model.

The resultant model is represented in Fig. 11.

Before delving into the specifics of the model, it’s pivotal to understand its structure and how to interpret the outcomes of the decision tree. Given that our model is a classification tree, it provides a structured framework that facilitates the derivation of decision rules. It’s worth noting that a detailed analysis of the model in this manner is uniquely possible for the decision tree approach. In the case of ensemble methods, the resultant models are inherently complex and challenging to visualize or interpret directly, given the multitude of individual models and their interactions.

Let us consider three decision rules with the highest coverage (support). Reading the tree from the top, if the distance covered by the patient during the 6 minute walking test is higher than 402.5 meter, AND:

- the exercise duration on a treadmill is lower or equal than 7.86 minutes, AND the task performance duration is higher than 3.95 minutes, AND patient level blood of potassium is lower or equal than 1.525 mEq/L THEN the patient’s NYHA class is two **(support: 29.7%; accuracy: 79.07%)**;
- the exercise duration on a treadmill is higher than 12.858 minutes, AND the task performance duration is lower or equal than 4.81 minutes, AND additionally the distance covered by the patient during the 6 minute walking test is higher than 497 meters THEN the patient’s NYHA class is one **(support: 12.2%, accuracy: 67.9%)**;
- the exercise duration on a treadmill is higher than 12.86 minutes, AND the task performance duration is higher than 4.81 minutes, AND patient level of blood peptide is higher than 645 pg/mL THEN the patient’s NYHA class is two **(support: 11,29%, accuracy: 71.4%)**;

On the contrary, now let us consider three decision rules with the lowest support, as well as with highest accuracy. Reading the tree from the top, if the distance covered by the patient during the 6 minute walking test is lower or equal than 402.5 m, AND:

- the exercise duration on a treadmill is higher than 12.11 minutes, AND additionally the distance covered by the patient during the 6 minute walking test is lower or equal than 275 m, AND a patient is treated with digoxin THEN the patient’s NYHA class is four **(support: 0.9%, accuracy: 100%)**;
- the exercise duration on a treadmill is higher than 12.11 minutes, AND additionally the distance covered by the patient during the 6 minute walking test is higher than 275 m, AND a patient level of blood peptide is between 393.35 and 615.5 pg/mL THEN the patient’s NYHA class is three **(support: 0.9%, accuracy: 100%)**;

With a different first premise, the third decision rule with the lowest support states, that, if the distance covered by the patient during the 6 minute walking test is higher than 402.5 meter, AND the exercise duration on a treadmill is between 7.86 and 12.858 min, AND the duration needed to complete the task is lower or equal 3.95 min, THEN the patient’s NYHA class is one **(support: 0.9%, accuracy: 100%)**.

We then determined the accuracy of the model by calculating the mean accuracy over the ten cross-validation iterations. The model showed an accuracy of 0.7628 with a standard deviation of 0.0742 (7.42%). Such a high value indicates significant variability in accuracy scores across different validation folds, suggesting that the model’s performance may not be entirely stable. Such a level of variability could potentially affect the reliability and consistency of the model’s predictions, introducing uncertainty into its accuracy. It’s important to interpret reported accuracy with caution given the level of variability observed during cross-validation.

The model’s accuracy of 0.7628 corresponds to a 76.28% probability that the model will make correct class predictions. Table 18 shows the confusion matrix for our decision tree model. The bolded numbers in the matrix represent the True Positive (TP) counts, which indicate correctly predicted instances for each respective class. Parenthetical values represent the proportion of instances of the actual class, regardless of the model’s prediction accuracy for that class.

**Table 18:** Confusion matrix for Decision Tree.

Predicted/Actual	NYHA I	NYHA II	NYHA III	NYHA IV
NYHA I	**57** (**74.03%**)	26 (11.71%)	3 (2.4%)	0
NYHA II	18 (23.37%)	**190** (**85.59%**)	42 (33.6%)	0
NYHA III	2 (2.6%)	6 (2.7%)	**80** (**64%**)	6 (60%)
NYHA IV	0	0	0	**4** (**40%**)

In terms of class-specific performance, NYHA II stands out with a commendable score of 85.59%. NYHA I and NYHA III classes followed closely with scores of 74.03% and 64% respectively. However, NYHA IV, despite its lower representation, lagged behind with a performance score of 40%.

Subsequently, we derived metrics like precision, recall, and the F1 score from the confusion matrix. These results are tabulated in Table 19.

**Table 19:** Evaluation metrics for Decision Tree model.

Class	Precision	Recall	F1-score
NYHA I	0.66	0.74	0.70
NYHA II	0.76	0.86	0.81
NYHA III	0.85	0.64	0.73
NYHA IV	1.0	0.40	0.57

The dataset prominently features the NYHA II class, which achieved the highest F1-score of 0.81, signifying excellent classification precision and recall. On the contrary, NYHA IV, being the least represented class, had the lowest F1-score of 0.57. This score, when juxtaposed with the others, is comparatively lower. The F1-scores for NYHA I and NYHA III were 0.70 and 0.73, respectively, indicating reasonable performance for these classes.

Table 20 presents a delineation of features based on their importance scores.

**Table 20:** Feature importances in Decision Tree model.

Feature	Importance
6MWT.DIST	0.402410
CPX.TIME	0.222276
EXERCISE1	0.135803
BNP	0.108318
HB	0.033999
K	0.032225
DIGOX	0.023390
DEATH?	0.013112
AGE	0.000000
MR	0.000000
QOL	0.000000
AF	0.000000
DM	0.000000
KIDNEY.DIS	0.000000
DIUR	0.000000

From the table the feature 6MWT.DIST, representing the distance covered in a 6-minute walking test, emerges as the most influential, followed by CPX.TIME and EXERCISE1. However, it’s crucial to note that the several features, such as AGE, MR, QOL, AF, DM, KIDNEY.DIS, and DIUR, have been assigned an importance score of zero. This suggests that they did not contribute to the model’s decision-making process. As a result, these variables are conspicuously absent in the decision tree visualized in the Fig. 11, underscoring the model’s ability to prioritize and discern between the variables based on their relevance.

In summary, the decision tree model demonstrated a commendable average accuracy of 76.28%, with particular prominence in the NYHA II class, while certain features such as 6MWT.DIST significantly influenced its predictions.

### Random Forest model

Owing to the intricate nature of the Random Forest model and the fact that it comprises multiple decision trees, a direct visualization of the model isn’t practical. The model showcased a performance metric of 0.9677, signifying a 96.77% probability of accurately predicting class outcomes. The confusion matrix associated with our Random Forest model is detailed in Table 21.

Due to the complexity of the Random Forest model and the fact that it consists of multiple decision trees, a direct visualization of the model is not practical. The model had a performance metric of 0.9677, meaning that it had a 96.77% probability of accurately predicting class outcomes. The observed standard deviation for this model was approximately 0.0295 (2.95%), indicating a low level of variability in accuracy scores across different validation folds. This suggests that the model is stable during predictions. The confusion matrix associated with our Random Forest model is detailed in Table 21.

**Table 21:** Confusion matrix for Random Forest model.

Predicted/Actual	NYHA I	NYHA II	NYHA III	NYHA IV
NYHA I	**73** (**94.8%**)	0	0	0
NYHA II	4 (5.2%)	**222** (**100%**)	10 (8%)	0
NYHA III	0	0	**115** (**92%**)	0
NYHA IV	0	0	0	**10** (**100%**)

Significant values are in [bold].

Delving into the Random Forest model’s confusion matrix reveals the excellent performance of the model. The NYHA I class boasts a 94.8% accuracy, with only a minor portion misclassified as NYHA II. Meanwhile, the NYHA II class stands out with a flawless 100% accuracy, althrough it occasionally misclassifies 8% of NYHA III instances. The NYHA III class itself performs commendably with 92% accuracy. Finally, the NYHA IV class also achieves a perfect 100% accuracy. Overall, the Random Forest model displays outstanding precision across all classes.

The performance metrics of the Random Forest model are detailed in Table 22

**Table 22:** Evaluation metrics for Random Forest mode.

Class	Precision	Recall	F1-score
NYHA I	1.0	0.95	0.97
NYHA II	0.94	1.0	0.97
NYHA III	1.0	0.92	0.96
NYHA IV	1.0	1.0	1.00

The evaluation metrics for the Random Forest model showcase its exemplary performance. The model achieved perfect precision across all classes. NYHA I demonstrated a recall of 0.95, leading to an F1-score of 0.97. The NYHA II class achieved an F1-score of 0.97 with a recall of 1.0. The NYHA III class, while maintaining a perfect precision, reported a recall of 0.92 and an F1-score of 0.96. The less frequent NYHA IV class secured perfect scores in all three metrics, underscoring the model’s robust classification abilities across diverse classes.

In addition, to examine the variation in the performance of individual features in our model, we evaluated the contribution of each feature to the classification task. By design, these scores are used to determine the relative importance of each feature in a data set and can be used to select relevant features for use in building a predictive model, while reducing dimensionality and noise in the data and improving overall model interpretability and performance^31^. While there are several approaches to this, the Random Forest model available in the Scikit-learn library calculates feature importance scores by averaging these scores across all decision trees in the ensemble^7^.

Table 23 presents the feature importance scores for the Random Forest model.

**Table 23:** Feature importances in Random Forest model.

Feature	Importance
6MWT.DIST	0.159156
CPX.TIME	0.140504
EXERCISE1	0.114844
BNP	0.102997
QOL	0.093678
K	0.090964
AGE	0.081295
HB	0.075023
MR	0.039724
DIUR	0.020477
DEATH?	0.018700
DM	0.016911
AF	0.016728
DIGOX	0.016259
KIDNEY.DIS	0.012739

In our Random Forest model, much like the Decision Tree model, the 6MWT.DIST attribute was identified as the most pivotal, boasting an importance score of 0.159156, signifying its crucial role in determining the target class. This was closely followed by CPX.TIME and EXERCISE1, with importance scores of 0.140504 and 0.114844 respectively. The influence of the other features was relatively less pronounced in the model’s decision-making process.

To conclude, the Random Forest model has showcased remarkable proficiency in categorizing, managing each class with notable precision and accuracy.

### Voting Classifier model

Analogous to the Random Forest model, the Voting Classifier is another ensemble technique, making its direct visualization impractical. This model registered an impressive accuracy rate of 99.54%, indicating a high likelihood of making correct class predictions. The observed standard deviation for this model was approximately 0.0092 (0.92%), which is a small value indicating minimal variability in accuracy scores across different validation folds. Such a low value suggests that the model exhibits considerable stability in its predictions.

The corresponding confusion matrix for the Voting Classifier model can be found in Table 24.

**Table 24:** Confusion matrix for Voting Classifier model.

Predicted / Actual	NYHA I	NYHA II	NYHA III	NYHA IV
NYHA I	**76** (**98.7%**)	0	0	0
NYHA II	0	**222** (**100%**)	0	0
NYHA III	1 (1.3%)	0	**125** (**100%**)	1 (10%)
NYHA IV	0	0	0	**9** (**90%**)

The model exhibits remarkable accuracy for the NYHA I and NYHA II classes, achieving 98.7% and a perfect 100% respectively. The NYHA III class also performs outstandingly, with a 100% accuracy rate. The NYHA IV class, on the other hand has a relatively lower accuracy rate of 90%, indicating a minor misclassification towards the NYHA III class. Overall, the Voting Classifier model demonstrates a high degree of precision across all the classes, reinforcing its reliability.

The evaluation metrics for the Voting Classifier model are presented in Table 25.

**Table 25:** Evaluation metrics for Voting Classifier model.

Class	Precision	Recall	F1-score
NYHA I	1.0	0.99	0.99
NYHA II	1.0	1.0	1.0
NYHA III	0.98	1.0	0.99
NYHA IV	1.0	0.9	0.95

The Voting Classifier model, being an ensemble method, calculates feature importance scores by averaging the significance assigned by all the individual models within the ensemble. This methodology ensures a balanced representation of feature influence. The detailed breakdown of these averaged feature importance scores is presented in Table 26.

**Table 26:** Feature importances for Voting Classifier model.

Feature	Importance
6MWT.DIST	0.469182
CPX.TIME	0.271016
EXERCISE1	0.074665
BNP	0.055366
K	0.033218
AGE	0.023427
HB	0.021363
MR	0.012500
QOL	0.010544
DEATH?	0.008536
DIGOX	0.008433
DIUR	0.004673
KIDNEY.DIS	0.003629
DM	0.002711
AF	0.000739

The Voting Classifier model, similar to both the Random Forest and Decision Tree models, emphasizes the 6MWT.DIST attribute as the most significant feature. This metric, representing the distance covered during a six-minute walk test, consistently emerges as a paramount determinant across the ensemble methods, with an importance score of 0.469182 in the Voting Classifier model. This consistency in feature importance underscores the attribute’s critical role in the classification process. Following 6MWT.DIST, CPX.TIME and EXERCISE1 are the next most influential attributes with scores of 0.271016 and 0.074665, respectively. The other features, while contributing, have a reduced impact on the model’s decision-making.

The Voting Classifier model stands out as a powerful ensemble technique, exhibiting exceptional precision and accuracy. With an impressive accuracy rate of 99.54%, it excels in classifying target classes, cementing its position as the most trustworthy model in our study.

### Comparison of classification models

In our study, we developed three classification models: Decision Tree, Random Forest, and the Voting Classifier.

The Table 27 below presents the accuracy of each model across different NYHA classes and average. The values highlighted in bold represent the best accuracy for the respective column.

**Table 27:** Accuracy of classification models for each NYHA class and average accuracy.

Model	NYHA I	NYHA II	NYHA III	NYHA IV	Average
Decision Tree	74.03%	85.59%	64%	40%	76.28%
Random Forest	94.8%	**100%**	92%	**100%**	96.77%
Voting Classifier	**98.7%**	**100%**	**100**%	90%	**99.54%**

One of the defining features of the Decision Tree model is its inherent clarity and ability to be interpreted. This allows for straightforward visualization and comprehension of its decision-making pathways. The ability to visually dissect the model provides immediate understanding of its decision logic. Although the model has an average accuracy of 76.28%, it demonstrates notable proficiency in classifying the NYHA II category.

In contrast, the Random Forest model, an ensemble of multiple decision trees, offers a more complex but powerful approach. Although we can’t visualize its intricate workings as we can with the Decision Tree, its performance metrics are outstanding. With an average accuracy of 96.77%. Among the classes, it delivers near-perfect accuracy for the NYHA I and NYHA II classes and a perfect F1-score for NYHA IV.

The Voting Classifier model emerges as the epitome of ensemble techniques. By leveraging insights from a myriad of models, it achieves a stellar accuracy rate of 99.54%. The precision this model exhibits across all classes is remarkable.

Upon comparison of the employed classification algorithms, the Voting Classifier emerges as the most empirically robust model, exhibiting unparalleled accuracy. This is closely trailed by the Random Forest model, which also exhibits significant efficacy. The Decision Tree, though not achieving comparable quantitative accuracy metrics, offers a qualitative advantage, furnishing critical interpretative insights.

Delving into class-specific performances provides further granularity to our analysis. For the NYHA I classification, the Voting Classifier model manifests superior efficacy, both in terms of the F1-score and an accuracy rate of 98.7%. In the context of the NYHA II classification, both the Random Forest and the Voting Classifier models exhibit exemplary performance, achieving an impeccable accuracy of 100%. For the NYHA III class, the Voting Classifier model retains its dominance, achieving an accuracy of 100%. In the classification of the NYHA IV class, the Random Forest model exhibits impeccable precision with a 100% accuracy rate, whereas the Voting Classifier, while highly effective, records an accuracy of 90%.

In summation, while each model possesses its unique strengths and utility, the empirical results favor the Voting Classifier in terms of average accuracy and precision across the classifications.

## Discussion

The novelty of this study lies in investigating different lifestyle factors that significantly contribute to predicting heart failure. In particular, this involves a detailed examination of the correlation and feature importance for particular groups. In addition, we embarked on a detailed exploration of the dataset, emphasizing a discerning attribute selection process to ensure the most relevant features were incorporated. Through the use of advanced ensemble techniques and predictive modeling, we were able to harness the potential of the dataset to its fullest.

The decision tree model, despite its apparent simplicity, provided crucial insights with a transparency that allowed us to understand its decision-making process. With an accuracy of 76.28%, it demonstrated that even straightforward models can be employed effectively for clinical decision support.

The ensemble techniques, namely the Random Forest and the Voting Classifier models, showcased the power of combining multiple models for enhanced prediction accuracy. The models achieved high accuracy of 96.77% and 99.54% respectively. These models, in particular, has the potential to assist cardiologists by streamlining the NYHA class assignment process, thus facilitating quicker and more precise treatment decisions for patients. This expedited classification not only ensures the deployment of the correct treatment in a timely manner but can also be pivotal in saving patients’ lives.

In comparison with other studies, the results obtained seem satisfactory in terms of predictive accuracy. However, it could be argued that a larger sample size would provide more reliable results. Indeed, this is a major limitation of our study and further research is needed to better understand and confirm our findings. Moreover, even with such promising results, our aim is to continuously refine and optimise the models developed. Therefore, our future directions include collecting more data, especially on the under-represented NYHA IV class, to obtain a more balanced data set. In addition, by re-examining the attributes and introducing feature engineering based on expert feedback, we hope to uncover deeper patterns within the data.

Note that there is no information in our data sample on the uptake of neprilysin inhibitors, a new class of drugs used to treat hypertension and heart failure. Because neprilysin inhibition generally resulted in a modest increase in blood peptide (BNP) levels as assessed by each assay. However, while some assays revealed no increase and others showed a decrease, this lack of information is another limitation of our study.

It should be emphasized that heart failure is a clinical diagnosis established by the presence of current or past characteristic symptoms. Such a disorder, in which the heart is unable to pump blood to the body at a rate commensurate with its needs, requires a comprehensive clinical diagnosis based on a careful medical history, physical examination, and imaging studies. While our models have demonstrated exemplary performance on our dataset, a real-world application will be the ultimate test of their efficacy. This necessitates external validation, pitting our models against independent datasets and juxtaposing their predictions with real-world decisions by specialists. This evaluation is crucial to ensure the practical applicability of our classification models.

## Conclusions

Due to certain lifestyle factors, such as smoking, obesity, lack of or limited physical activity, it is expected that there will be an increase in the number of patients with heart failure. in the number of people with heart failure. Finally, this study sets a promising precedent for future research in this area. The evident potential of predictive performance models to improve clinical decision making, coupled with continuous refinement and rigorous validation, signals a bright future for the application of machine learning in medicine. In our opinion, the models developed could be very useful in their supporting role in clinical practice, as the use of a machine learning tool could reduce or even eliminate the bias in the physician’s assessment.

## Data Availability

Data available on reasonable request. Please contact correspondence author for access to dataset.
